# *Sox6* Up-Regulation by Macrophage Migration Inhibitory Factor Promotes Survival and Maintenance of Mouse Neural Stem/Progenitor Cells

**DOI:** 10.1371/journal.pone.0074315

**Published:** 2013-09-16

**Authors:** Shigeki Ohta, Aya Misawa, Véronique Lefebvre, Hideyuki Okano, Yutaka Kawakami, Masahiro Toda

**Affiliations:** 1 Division of Cellular Signaling, Institute for Advanced Medical Research, Keio University School of Medicine, Shinjuku-ku, Tokyo, Japan; 2 Department of Cellular and Molecular Medicine (NC10) Cleveland Clinic Lerner Research Institute, Cleveland, Ohio, United States of America; 3 Department of Physiology, Keio University School of Medicine, Shinjuku-ku, Tokyo, Japan; 4 Department of Neurosurgery, Keio University School of Medicine, Shinjuku-ku, Tokyo, Japan; University of Nevada School of Medicine, United States of America

## Abstract

Macrophage migration inhibitory factor (MIF) has important roles in supporting the proliferation and/or survival of murine neural stem/progenitor cells (NSPCs), but downstream effectors remain unknown. We show here that MIF robustly increases the expression of *Sox6* in NSPCs *in vitro*. During neural development, Sox6 is expressed in the ventricular zone of the ganglionic eminence (GE) of mouse brains at embryonic day 14.5 (E14.5), cultured NSPCs from E14.5 GE, and NSPCs in the subventricular zone (SVZ) around the lateral ventricle (LV) of the adult mouse forebrain. Retroviral overexpression of Sox6 in NSPCs increases the number of primary and secondary neurospheres and inhibits cell differentiation. This effect is accompanied with increased expression of *Hes1* and *Bcl-2* and Akt phosphorylation, thus suggesting a role for *Sox6* in promoting cell survival and/or self-renewal ability. Constitutive activation of the transcription factor Stat3 results in up-regulation of *Sox6* expression and chromatin immunoprecipitation analysis showed that MIF increases Stat3 binding to the *Sox6* promoter in NSPCs, indicating that Stat3 stimulates *Sox6* expression downstream of MIF. Finally, the ability of MIF to increase the number of primary and secondary neurospheres is inhibited by *Sox6* gene silencing. Collectively, our data identify Sox6 as an important downstream effector of MIF signaling in stemness maintenance of NSPCs.

## Introduction

The Sox protein family of transcription factors has been identified as a major group of developmental regulators in vertebrates and invertebrates [1]. Sox transcription factors induce or suppress progenitor cell properties, such as proliferation and multipotentiality, or initiate differentiation programs by activating the expression of cell type-specific genes. The Sox family is comprised of 20 genes, classified into 8 groups (A to H), which encode transcription factors with a high-mobility-group (HMG) box DNA-binding domain highly similar to that of the sex-determining region (Sry) protein [2]. Sox2, which is a SoxB protein, is a mandatory maintenance factor in neural/stem progenitor cells (NSPCs) in fetal and adult mouse brains [3],[4],[5]. However, the detailed function of most Sox genes in the developing nervous system and in NSPCs remains to be elucidated.

Sox6 belongs to the SoxD family, along with Sox5 and Sox13. SoxD proteins harbor two highly conserved functional domains: the family-specific HMG box DNA-binding domain and a group-specific coiled-coil domain that mediates homodimerization [6]. They have no known transactivation or transrepression domain, but participate in transcriptional activation and repression by utilizing various cofactors to modulate cell proliferation, survival, differentiation, and terminal maturation in a number of mesoderm-, ectoderm-, and endoderm-derived cell lineages [7]. Sox6 contributes to erythropoiesis and chondrogenesis and Sox6 knockout mice die soon after birth, presumably from cardiac malformation [8], [9]. In addition, Sox6 inhibits terminal differentiation of oligodendrocytes [10] and contributes to the specification of diverse types of neurons *in vitro* and *in vivo* [11], [12], [13] and favors the differentiation of rat NSPCs *in vitro* towards the astrocyte rather than neuronal lineage [14]. However, the mechanisms underlying Sox6 expression and the exact functions of Sox6 in NSPCs remain underexplored.

We previously reported that *SOX6* is highly expressed in human gliomas [15] and that Macrophage migration inhibitory factor (MIF) supports the proliferation and/or survival of murine NSPCs *in vitro* [16]. Here we show that Sox6 is expressed in the ventricular zone of the ganglionic eminence (GE) of E14.5 mouse fetal brains, and in the subventricular zone (SVZ) around the lateral ventricle (LV) of mouse adult forebrains, where NSPCs are located. We also show that *Sox6* expression is increased by MIF in NSPCs *in vitro*, and that this effect is mediated by the transcription factor Stat3. Further, we demonstrate an important role for Sox6 in supporting the viability and self-renewal ability of NSPCs under the control of MIF.

## Materials and Methods

### Animals

All interventions and animal care procedures were performed in accordance with the Laboratory Animal Welfare Act, the Guide for the Care and Use of Laboratory Animals (National Institutes of Health, USA), and the Guidelines and Policies for Animal Surgery provided by the Animal Study Committee of the Keio University and were approved by the Animal Study Committee of Keio University (IRB approval number 12017-0). Pregnant C57BL/6J mice were purchased from Sankyo Labo Service (Tokyo, Japan). Sox6 knockout mice [9] were maintained on a 129 background.

### Neurosphere Culture

NSPCs were isolated from mouse E14.5 GEs and the cells were cultured as neurospheres [17] at a cell density of 50 cells/µl in neurosphere culture medium (NSP medium) consisting of neurobasal medium (Invitrogen, Carlsbad, CA, www.invitrogen.com) supplemented with B27 (Invitrogen), human recombinant (hr) EGF (20 ng/ml; Peprotech, Rocky Hill, NJ, www.peprotech.com), and hrFGF2 (10 ng/ml; Peprotech). Neurosphere formation assays were performed as described previously [16]. In the experiments using NSPCs, mouse recombinant MIF (R&D systems, Mineapolis, MN, www.rndsystems.com) and MIF inhibitor ISO-1 (Calbiochem, La Jolla, CA, www.merckmillipore.com) were used. Human NSPCs were cultured as reported by Hattori Y et al., [18]. The study using human NSPCs was carried out in accordance with the principles of the Helsinki Declaration, and the Japan Society of Obstetrics and Gynecology. Approval to use human fetal neural tissues was obtained from the ethical committees of both Osaka National Hospital and Keio University. Written informed consent was obtained from all parents through routine legal terminations performed at Osaka National Hospital. Human glioma cells (SF126) and human fibroblast cells (TIG-118) were obtained from the Japanese Collection of Research Bioresources (JCRB, Osaka, Japan, www.cellbank.nibio.go.jp), and human glioma cells (U87MG) were obtained from the American Type Culture Collection (ATCC, Manassas, VA, www.atcc.org).

### RNA Extraction and Quantitative (q) RT-PCR

Total RNA was isolated from tissues or cultured cells using TRIZOL (Invitrogen). Total RNA (0.5 μ g) was subjected to the cDNA Synthesis using ReverTra Ace^®^ qPCR RT Master Mix with gDNA Remover (Toyobo, Osaka, Japan, www.toyobo.co.jp). Quantitative RT-PCR analysis was performed with a FastStart Universal SYBR Green Master (Roche, Tokyo, www.app.roche-biochem.jp), using the ABI prism 7900 HT Sequence Detection System (Applied Biosystems, Life Technologies, Carlsbad, www.appliedbiosystems.com). The PCR conditions were as follows: 1 cycle of 5 min at 95°C, followed by 40 cycles of 95°C for 30 sec, 60°C for 60 sec, and 72°C for 30 sec. PCR reactions were performed in triplicate. Relative gene expression levels were determined using the ΔΔ*C*t-method. GAPDH mRNA levels were used as the internal normalization control. The primer sequences are listed in Table.S1 and the sequence for *Hes1*, *Hes3*, and *GAPDH* primers were described in the previous study [16].

### Retrovirus and Lentivirus Production

The mouse *Sox6* cDNA (IRAKp961F19144Q, RZPD, Berlin, Germany, www.rzpd.de) was subcloned into the pMX vector [19] and pMX-Stat3-C was obtained from Addgene (Cambridge, MA, www.addgene.org). To construct short hairpin RNA (shRNA)-expressing retroviral vectors, oligonucleotides targeting the coding sequence of *Sox6* gene (CAGCCCUGUAACUCAAGUU) and luciferase (Clontech, Mountain View, CA, www.clontech.com) were inserted into pSIREN vector (Clontech). A self-inactivating vector plasmid containing DNA fragments of human *SOX6* promoter (−425∼+34) and Venus cDNA (gifted from Dr. Miyawaki A) were constructed based on the modified CS-CDF-CG-PRE vector (gifted from Dr. Miyoshi H) as described in a previous report [20]. Recombinant lentiviruses were produced by the shSox6 lentivirus vector (Validated clone, TRCN00000085945, Sigma, St. Louis, MO, www.sigmaaldrich.com) or control shRNA vector (SHC002, Sigma). The retrovirus and lentivirus production were performed as described previously [16].

### Luciferase Assay

NSPCs were plated at a density of 5,000 cells/well on 96-well plates. 100 ng of a pGL3 reporter plasmid and 1 ng of a pRL-TK (Promega, Tokyo, Japan, www.promega.co.jp) internal control plasmid were co-transfected using the X-tremeGENE HP DNA Transfection Agent (Roche) according to the manufacturer’s instructions. After 24 h of transfection, the cells were treated with MIF (400 ng/ml). Luciferase activity was measured after 24 h using the Dual-Glo Luciferase Assay System (Promega). Relative luciferase activity was calculated by dividing the firefly luciferase activity of the constructs by the Renilla luciferase activity of the tyrosine kinase promoter, pRL-TK (Promega).

### Chromatin Immunoprecipitation

Cells were subjected to chromatin immuneprecipitation with the ChIP-IT kit (Active Motif, Carlsbad, CA, www.activemotif.com) using anti-Stat3 antibody (Cell Signaling technologies, Danvers, MA, www.cellsignal.com) or normal rabbit IgG following the manufacturer’s protocol. After elution, samples were quantified using FastStart Universal SYBR Green Master and primers, which targets the upstream region of the starting point for Sox6 transcription (EpiTct ChIP qPCR Primers, GPM1053672(−)03A, SABiosciences, Frederick, MD, www.sabiosciences.com).

### Western Blot Analysis

Cell lysates were prepared using RIPA buffer (25 mM Tris–HCl, 150 mM NaCl, 1% NP-40, 1% sodium deoxycholate, and 0.1% SDS, pH 7.6) containing protease inhibitors (Cocktail Tablet; Roche). Lysates were centrifuged at 14,000×*g* for 15 min at 4°C, and the protein concentration of each sample was determined using a Bio-Rad protein assay kit (Bio-Rad, Tokyo, Japan, www.bio-rad.com) with bovine serum albumin as a standard. Identical amounts of protein were electrophoresed in 10% SDS-PAGE gels and transferred to a nitrocellulose membrane. Blots were blocked with Blocking One™ (Nacalai Tesque, Kyoto, Japan, www.nacalai.co.jp) at RT for 1 hr, then incubated with primary antibodies overnight at 4°C as follows: Akt (1∶1000), phospho-Akt (Ser473) (1∶1000; Cell Signaling technologies), Bcl2 (1∶1000; MBL, Nagoya, Japan, www.mbl.co.jp), Sox6 (1∶2000; Santa Cruz Biotechnology), and Actin (1∶5000; Sigma). After three washes in TBST (20 mM Tris–HCl, 150 mM NaCl, and 0.02% Tween-20, pH 7.4), the blots were incubated with the appropriate secondary antibodies conjugated with horseradish peroxidase (1∶4,000, anti-rabbit and anti-mouse; GE Healthcare, Tokyo, Japan, www.japan.gehealthcare.com) for 1 h at room temperature. Signals were detected with ECL-Plus Substrate (GE Healthcare) and exposed to Hyperfilm (GE Healthcare). The intensity of the bands was measured by densitometric analysis using NIH ImageJ software (http://imagej.nih.gov/ij).

### Cell Proliferation and Apoptosis Assay

Cell viability were assessed using Cell Titer-Glo Luminescent Cell Viability Assay kits (Promega) and a luminometer (EnVision™ multilabel reader, Perkin Elmer, Waltham, MA, www.perkinelmer.com). Cell apoptosis was assessed using Caspase-Glo 3/7 Assay Kits (Promega). In both assays, single cells dissociated from neurospheres were seeded onto 96-well plates at a density of 5×10^3^ cells/well, and caspase activity was assayed 4 days post-infection.

### Immunocytochemistry and Immunohistochemistry

Embryonic brains were removed and fixed in 4% paraformaldehyde (PFA) in 0.1 M phosphate-buffered saline (PBS), cryoprotected in 30% sucrose solution in PBS, and embedded in O.C.T. compound (Sakura Finetek, Tokyo, Japan, www.sakura-finetek.com). Adult mice were killed by anesthetic overdose and perfused transcardially with 4% PFA in PBS, pH 7.2. Brains were postfixed in the perfusion solution overnight at 4°C, then cryoprotected for at least 24 h in 30% sucrose in PBS and embedded as above. Brain blocks were sectioned in the appropriate plane in 14 µm slices. After blocking with 10% goat normal serum in 0.1 M PBS, brain slices were incubated in 5% goat normal serum in 0.1 M PBS + 0.3% Triton X-100 with the following primary antibodies: rabbit anti-Sox6 (1∶100; Santa Cruz Biotechnology, Santa Cruz, CA, www.scbt.com; 1∶100, Abcam, Cambridge, MA, www.abcam.com), mouse anti-Nestin, (1∶5; Developmental Studies Hybridoma Bank, Iowa City, IA, dshh.biology.uiowa.edu), mouse anti-Sox2 (1∶100; R&D systems), mouse anti-Ascl1 (1∶100; BD Bioscience, Bedford, MA, www.bdbiosciences.com), mouse anti-GFAP (1∶200; Sigma), and rat anti-BrdU (1∶100; Abcam). Application of the primary antibodies was followed by incubation of the brain slices with secondary antibodies labeled with Alexa Fluor 488, and 568 (1∶400; Invitrogen). Short time BrdU chase experiments were performed in the adult mouse brain following a method that has been reported previously [17]. For immunocytochemical studies, cells were fixed with PBS containing 4% PFA for 20 min at room temperature, and the cells were subjected to immunofluorescence staining using the following primary antibodies: rabbit anti-Sox6 (1∶200; Santa Cruz Biotechnology), mouse anti-Nestin (1∶5; DSHB), mouse anti-β-tubulin type III (TuJ1) (1∶1000; Sigma), mouse anti-CNPase (1∶250; Sigma), and rabbit anti-GFAP (1∶400; Biomedical Technologies, Stoughton, MA, www.btiinc.com). After PBS washes, antibody binding was visualized using either Alexa Fluor 488 or 568-conjugated secondary antibodies (Invitrogen), and the nuclei were stained with either DAPI or TO-PRO-3 (Invitrogen). In differentiation assays, single dissociated cells of cultured neurospheres were plated on poly-L-lysine coated glass slips at a density of 2×10^5^ cells/cm^2^ in NSP medium without growth factors for 5 days, and then subjected to immunocytochemical analysis. In the analyses, at least 10 different viewing fields were counted using confocal microscopy (Zeiss, Tokyo, Japan, www.zeiss.co.jp).

### Flow Cytometry

Flow cytometric analyses were performed using Flow cytometry EPICS-*XL* (Beckman Coulter, Tokyo, Japan, www.beckmancoulter.co.jp). Cells were isolated using a EPICSAltra cell sorter (Beckman-Coulter).

### Statistical Analysis

All values are expressed as mean±S.D. Student’s *t* tests were used to determine the statistical significance of differences between groups (*P<0.05, **P<0.01).

## Results

### Sox6 is a Downstream Target of MIF Signaling in NSPCs

**Figure 1:**
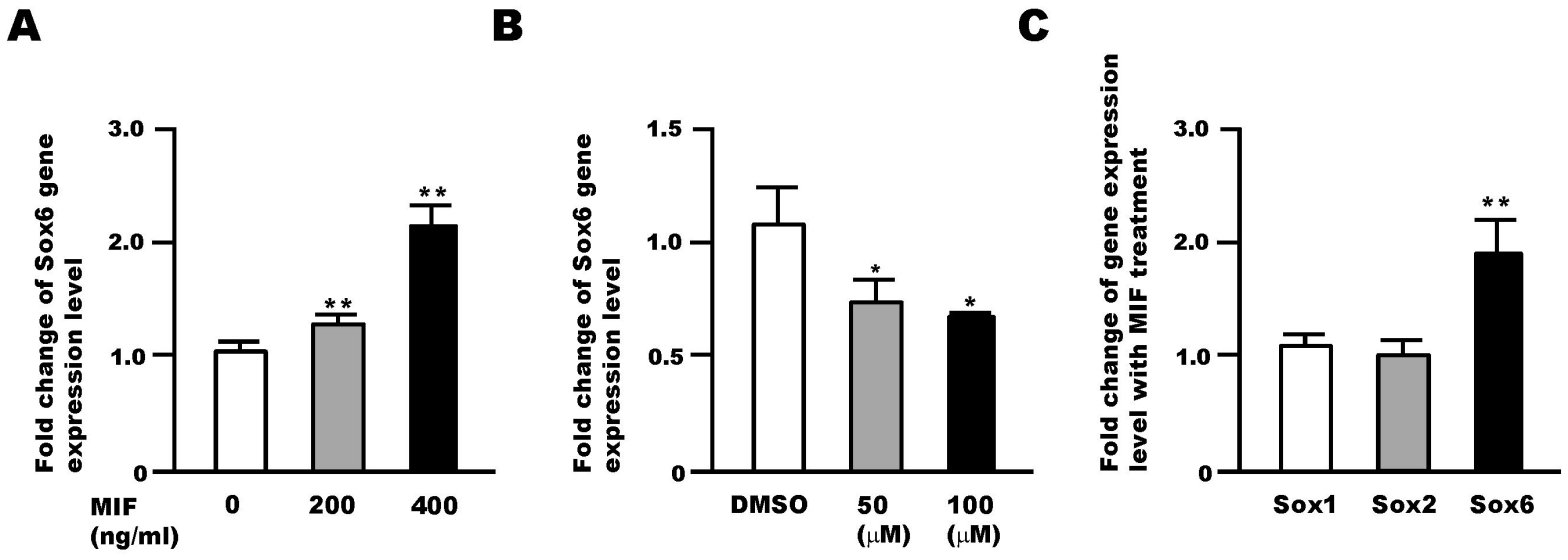
Sox6 functions downstream of MIF in NSPCs. (A) MIF treatment for 24 h increases *Sox6* gene expression. (B) MIF antagonist (ISO-1) treatment for 24 h decreased *Sox6* gene expression in NSPCs. (C) Changes in Sox gene expression levels in NSPCs following MIF treatment (400 ng/ml) for 24 h. Data are derived from three independent experiments. Error bars indicate S.D. values; *P<0.05, **P<0.01 versus control; Student’s *t*-test.

Changes in the expression levels of the Sox genes upon treatment of NSPCs with MIF were examined by qRT-PCR. MIF treatment increased the RNA level of *Sox6* in NSPCs (Fig. 1A). In addition, cell treatment with ISO-1, a MIF inhibitor, led to a decrease in *Sox6* RNA level in a dose-dependent manner (Fig. 1B). Interestingly, MIF treatment increased the RNA level of *Sox6*, but not that of *Sox1* and *Sox2* (Fig. 1C). Together, these results suggest that the *Sox6* gene is a downstream target of MIF signaling in NSPCs.

### Sox6 is Expressed in Mouse Embryonic Brain NSPCs

**Figure 2:**
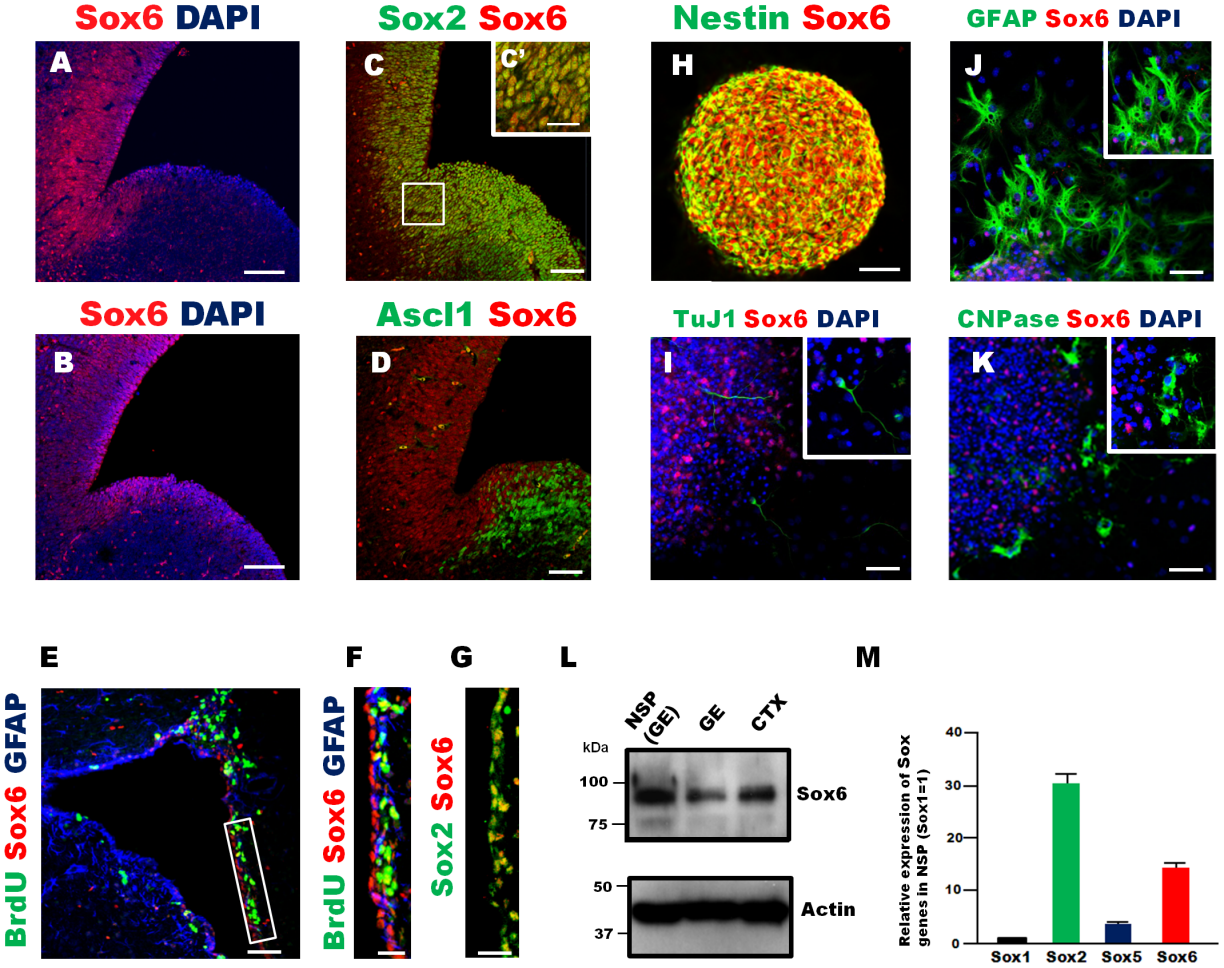
Sox6 expression in NSPCs. (A, B) Expression of Sox6 in E14.5 lateral ganglionic eminences (LGE) labeled with different rabbit anti-Sox6 antibodies (A, Abcam; B, Santa Cruz Biotechnology). (C) Immunohistochemistry of Sox6-positive cells co-labeled with Sox2 in E14.5 LGE. (D) Sox6-positive cells were located more towards the ventricle compared to cells expressing Ascl1 in E14.5 GE. (E) Sox6 expression in the SVZ of the adult mouse (6 weeks of age) forebrain. Sox6 was co-labeled with GFAP and short time-labeled BrdU. (F) Enlarged image of the boxed area in E. (G) Immunohistochemistry of the adult mouse forebrain using Sox6 and Sox2 antibodies. Most Sox6-positive cells in the SVZ co-labeled with Sox2, marker for NSPCs. (H) Immunocytochemistry of neurospheres using Sox6 and Nestin antibodies. Most Sox6-positive cells in the neurospheres generated from E14.5 GE were co-labeled with Nestin, a marker for NSPCs. (I–K) Immunocytochemistry of differentiated neural cells generated from E14.5 GE-derived neurospheres 5 days after *in vitro* differentiation. Although a small number of Sox6-positive cells was observed in each differentiated cell types identified by the following markers: TuJ1(I), GFAP (J), and CNPase (K), most of Sox6-positive cells were not stained with the differentiation markers. Scale bar: 100 µm (A, B), 50 µm (C–E, H–K), 20 µm (C’, F, G). (L) Western blot analysis showing Sox6 protein expression in NSPCs generated from E14.5 GE (NSP), E14.5 GE (GE), and E14.5 cortex (CTX). (M) Relative gene expression levels of *Sox1*, *Sox2*, *Sox5*, and *Sox6* in E14.5 GE-derived neurospheres. Data show a representative data from three independent experiments.

The expression of the Sox6 protein in the mouse embryonic brain at E14.5 was examined by immunofluorescence. Sox6 was expressed in the ventricular zone of the cortex and the dorsal part of the lateral ganglionic eminence (LGE), as previously shown [12] (Fig. 2A and B). The Sox6-positive cells also expressed Sox2, a known marker of NPSCs (Fig. 2C), and these cells were located more towards the ventricle compared to cells expressing Ascl1, a marker of neural progenitor cells, in the E14.5 GE (Fig. 2D). In the adult mouse forebrain, Sox6 was expressed in the subventricular zone (SVZ) (Fig. 2E). Some Sox6-expressing cells were positive for BrdU after short-term labeling, indicating that they were actively proliferating, and they were also positive for Sox2, proving that they corresponded to NSPCs (Fig. 2, E–G). In the sub-granular zone (SGZ) of the adult mouse hippocampus, Sox6 expression coincided with BrdU labeling expression (data not shown), indicating that Sox6 was expressed in NSPCs in the adult as it is in the embryo. Next, we generated neurospheres from E14.5 GEs and observed co-expression of Sox6 with Nestin, a marker of NSPCs (Fig. 2H). To examine the expression of Sox6 in differentiated cells, neurospheres were cultured without growth factors for 5 days *in vitro* (DIV). Besides a few exceptions, Sox6 expression was detected only in cells negative for differentiation markers, namely TuJ1 (neurons), GFAP (glia), and CNPase (oligodendrocytes) (Fig. 2, I–K). This result is consistent with a report showing that Sox6 is highly expressed in oligodendrocyte progenitor cells (OPCs) but not in post-mitotic differentiating oligodendrocytes [10]. Sox6 protein expression in NSPCs from 14.5 GE was also confirmed by Western blot (Fig. 2L). Finally, we compared the expression level of *Sox6* to that of other *Sox* genes by qRT-PCR in NSPCs derived from E14.5 GE. The data clearly indicated that NSPCs contained higher levels of RNA for *Sox2* and *Sox6* than for their respective close relatives, *Sox1* and *Sox5* (Fig. 2M).

### Sox6 Supports Cell Survival and the Self-renewal Ability of NSPCs

**Figure 3:**
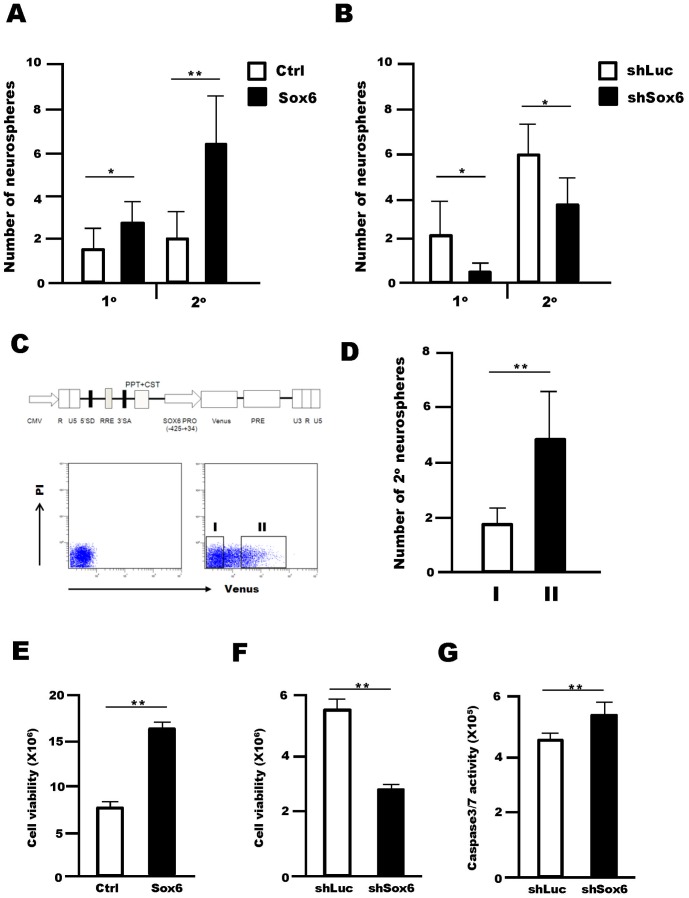
Sox6 increases survival and/or self-renewal ability of NSPCs. (A) In the primary neurosphere formation assay, single dissociated cells of neurospheres generated from E14.5 GE were plated onto a 96-well plate and infected with retroviruses encoding GFP (Ctrl) or Sox6 and GFP (Sox6). The cells were cultured in the presence of both EGF and FGF2. In the secondary neurosphere assay, neurospheres infected with a Sox6-expressing retrovirus or control for 5DIV were sorted as GFP-positive cells onto a 96-well plate and cultured in the presence of both EGF and FGF2. (B) Sox6 targeting using retroviral shRNA significantly attenuated the efficiency of both primary and secondary neurosphere formation. In the primary neurosphere assay, single dissociated neurospheres were infected with a retrovirus expressing either Luc-shRNA (shLuc) or Sox6-shRNA (shSox6). In the secondary neurosphere assay, neurospheres infected with a retrovirus expressing either Luc-shRNA or Sox6-shRNA for 5 DIV were dissociated into single cells and plated onto a 96-well plate for the culture in the presence of EGF and FGF2. (C) Schematic representation of the self-inactivating lentivirus vector expressing the Venus reporter gene under the control of the *SOX6* promoter. Flow cytometric analysis of neurospheres infected with lentivirus expressing the Venus reporter regulated by a *SOX6* promoter for 5 days. (D) Venus-positive cells (II) and Venus-negative cells (I) were sorted onto a 96-well plate and cultured in the presence of EGF and FGF2, and the number of secondary neurospheres was quantified. All neurosphere formation assays were performed at a low cell density (1 cell/µl). Data are representative of three independent experiments. Error bars indicate S.D. values. **P<0.01 versus control; Student’s *t*-test. (E) The viability of cells dissociated from neurospheres was assessed 5 days after infection with either a control retrovirus or a retrovirus expressing Sox6 using a CellTiter Glo Luminescent Cell Viability Kit. (F) *Sox6* targeting via retroviral shRNA significantly reduced NSPC growth compared to Luc-shRNA, as assessed using Cell Titer-Glo Assay Kit 4 days of post-infection. (G) *Sox6* targeting via retroviral shRNA led to an increase in caspase 3/7 activity in NSPCs 4 days after infection. Data show a representative data from three independent experiments Error bars indicate S.D. values; *P<0.05, **P<0.01 versus control; Student’s *t*-test.

To identify the function of Sox6 in NSPCs, we first performed neurosphere-forming assays, a commonly used method to measure NSPC self-renewal. Retrovirus-mediated Sox6 overexpression led to a 1.7±0.6 fold increase in the number of primary neurospheres, and 3.2±1.1 fold increase in the number of secondary neurospheres (Fig. 3A). In contrast, *Sox6* silencing by retroviral expression of shRNA-Sox6 in NSPCs attenuated the formation of primary and secondary neurospheres by 0.23±0.25 fold and 0.62±0.22 fold, respectively (Fig. 3B). This result was also supported in experiments using Sox6-null mice (Fig. S1). Changes in Sox6 protein levels in NSPCs by Sox6 overexpression and gene silencing were assessed by Western blot analysis (Fig. S2). Moreover, we performed the neurosphere-forming assay using a lentivirus-based Sox6 reporter system. A reporter plasmid was constructed using a self-inactivating lentivirus vector harboring the Venus reporter gene under the control of the human *Sox6* promoter region, which contains elements highly conserved in humans and mice in terms of tissue-specific expression [21] (Fig. 3C). Venus-positive cells (II) and Venus-negative cells (I) were sorted onto a 96-well plate and then cultured in the presence of EGF and FGF2, and the number of secondary neurospheres was assessed (Fig. 3D). The specificity of the reporter assay was confirmed in human cells and mouse NSPCs by showing a strong correlation between Venus activity and Sox6 protein level (Fig. S3). In this system, Venus-positive NSPCs formed 2.8±1.0 fold more secondary neurospheres than Venus-negative cells (Fig. 3D). Collectively, these data indicated that Sox6 supports the self-renewal ability of NSPCs *in vitro*. We then asked whether the *Sox6* promoter featured MIF responsive regions. The promoter contains two regions, called A-box (79 bp) and B-box (48 bp), which are 85 and 96% identical, respectively, in the rat, mouse, and human genomes [21]. NSPCs were transfected with luciferase reporters containing either a promoter fragment (517 bp) or tandem repeats of the A-box (4xA-box) or B-box (4xB-box) and were then treated with or without MIF (Fig. S4). The construct containing the A-box tandem repeats showed strong transcriptional activity, and this activity was increased 3.4±0.8 fold by MIF treatment, suggesting that the A-box contains MIF-responsive elements (Fig. S4). We also examined changes in NSPC viability upon Sox6 overexpression and silencing. Retrovirus-mediated overexpression of Sox6 in NSPCs increased cell viability by 2.1±0.12 fold 5 days after infection (Fig. 3E), whereas retrovirus-mediated silencing of Sox6 decreased cell viability by half (0.50±0.02 fold) 4 days after infection (Fig. 3F). Sox6 knockdown also led to increased caspase3/7 activity 4 days after infection, confirming that Sox6 acts as a survival factor in NSPCs (Fig. 3G, Fig. S5).

### Sox6 is a Maintenance Factor in NSPCs

**Figure 4:**
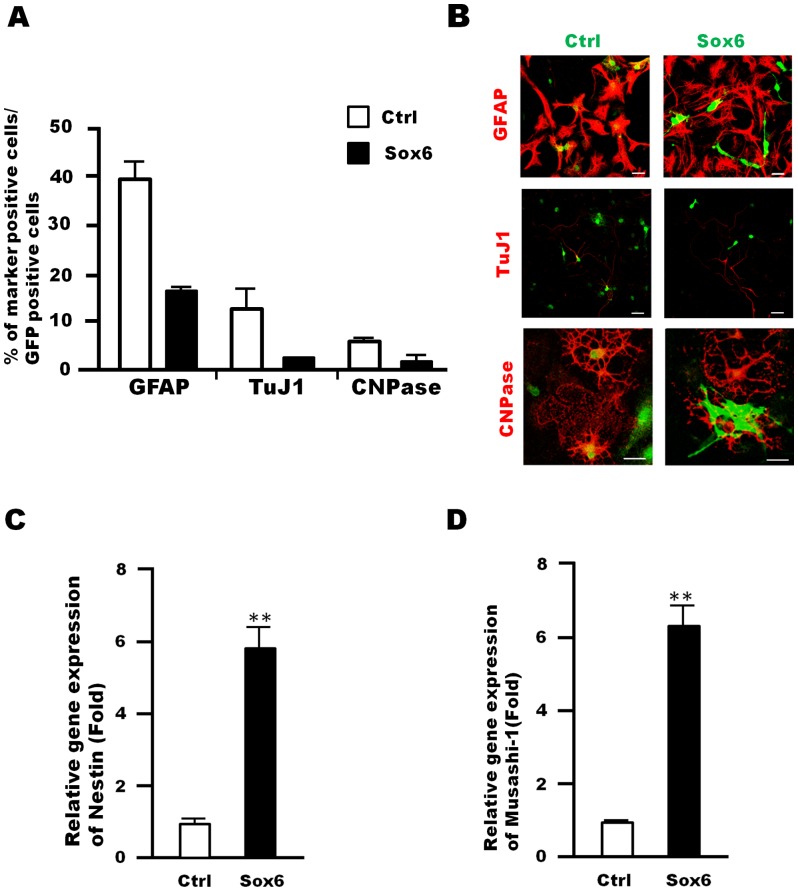
Sox6 overexpression decreased the multi-lineage differentiation potential in NSPCs. (A) Neurospheres infected with either a control retrovirus expressing GFP or a retrovirus expressing Sox6 and GFP were cultured for 5 days in the presence of EGF and FGF2. The neurospheres were allowed to dissociate and were then plated onto poly-L-ornithin-coated coverslips at a density of 2×10^5^ cells/cm^2^ and cultured for another 5 days without growth factors. Fully differentiated cells were fixed and subjected to immunocytochemical analyses. The number of GFP-positive cells that were co-labeled with a neuronal marker (TuJ1), an astrocyte marker (GFAP), or an oligodendrocyte marker (CNPase) was counted and the data were expressed as percentages of GFP-positive cells. The graph represents the average of two independent experiments. (B) Representative images of cells that differentiated from neurospheres infected with a control retrovirus expressing GFP (Ctrl) or a retrovirus expressing Sox6 and GFP (Sox6). Scale bar: 20 µm. (C, D) Retroviral Sox6 overexpression in NSPCs led to an increase in Nestin (C) and Musashi-1 (D) gene expression 5 days after infection. Data are representative of three independent experiments. Error bars indicate S.D. values; **P<0.01 versus control; Student’s *t*-test.

Changes in the multi-lineage differentiation potential of NSPCs were examined upon Sox6 overexpression. NSPCs infected with a Sox6-expressing retrovirus were cultured for 5 days, and then replated and cultured for 5 additional days in the absence of growth factors. Then, the number of cells differentiated into each neuronal cell type was assessed by immunocytochemistry. In this assay, only GFP-positive cells, i.e., infected by the retrovirus, were examined. Sox6 overexpression led to a decrease in the number of cells differentiated into neurons (TuJ1), astrocytes (GFAP), and oligodendrocytes (CNPase) (Fig. 4, A and B). In addition, the expression of *Nestin* and *Musashi-1*, which are known as NSPC markers [22],[23], was also upregulated by Sox6 overexpression (Fig. 4, C and D). Thus, Sox6 functions as a maintenance factor for NSPCs in vitro. Consistent with this experiment, the numbers of neurons, astrocytes and oligodendrocytes were increased in NSPC cultures from Sox6-null mice (Fig. S6).

### Downstream Signal of Sox6 in NSPCs

**Figure 5:**
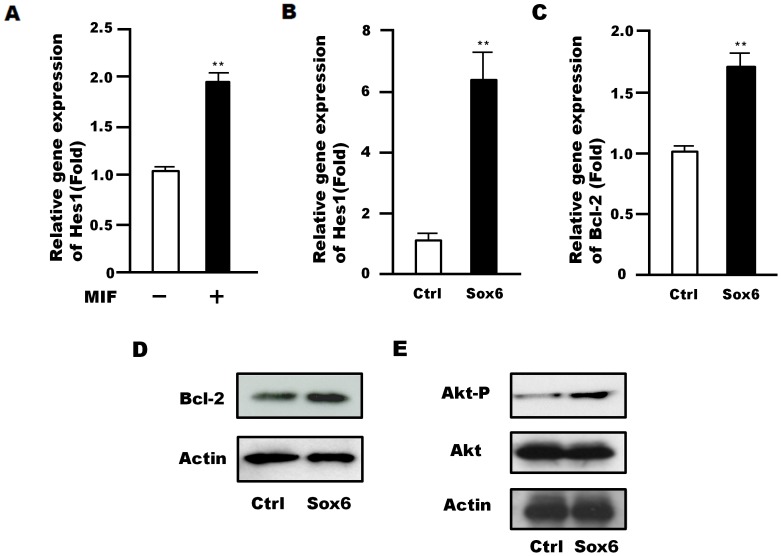
Analysis of signaling pathways downstream of Sox6 in NSPCs. (A) Changes in *Hes1* gene expression levels in NSPCs following MIF treatment (400 ng/ml) for 48 h. (B, C) Retroviral Sox6 overexpression in NSPCs led to an increase in *Hes1* (B) and *Bcl-2* (C) gene expression 5 days after infection. Data are representative of three independent experiments. Error bars indicate S.D. values; **P<0.01 versus control; Student’s *t*-test. (D) Retroviral overexpression of Sox6 increased Bcl-2 protein expression in NSPCs 2 days after infection. (E) Akt phosphorylation increased by Sox6 overexpression after 5 days of retroviral infection compared to controls. Data are representative images of three independent experiments.

Hes1 is a transcription factor acting downstream of Notch signaling and well known for its ability to maintain the stemness of NSPCs [24]. Interestingly, *Hes1* expression was increased upon treatment of NSPCs with MIF (Fig. 5A) as well as upon overexpression of Sox6 (Fig. 5B). Furthermore, expression of the *Bcl-2 *gene, which is important for cell survival and is regulated by MIF in NSPCs [16], also increased with Sox6 overexpression (Fig. 5C). This increase was confirmed at the protein level for Bcl-2 (Fig. 5D) and found to occur concomitantly with an increase in Akt phosphorylation (1.5±0.11 fold) (Fig. 5E).

### MIF-regulated Stat3 Controls Sox6 Gene Expression in NSPCs

**Figure 6:**
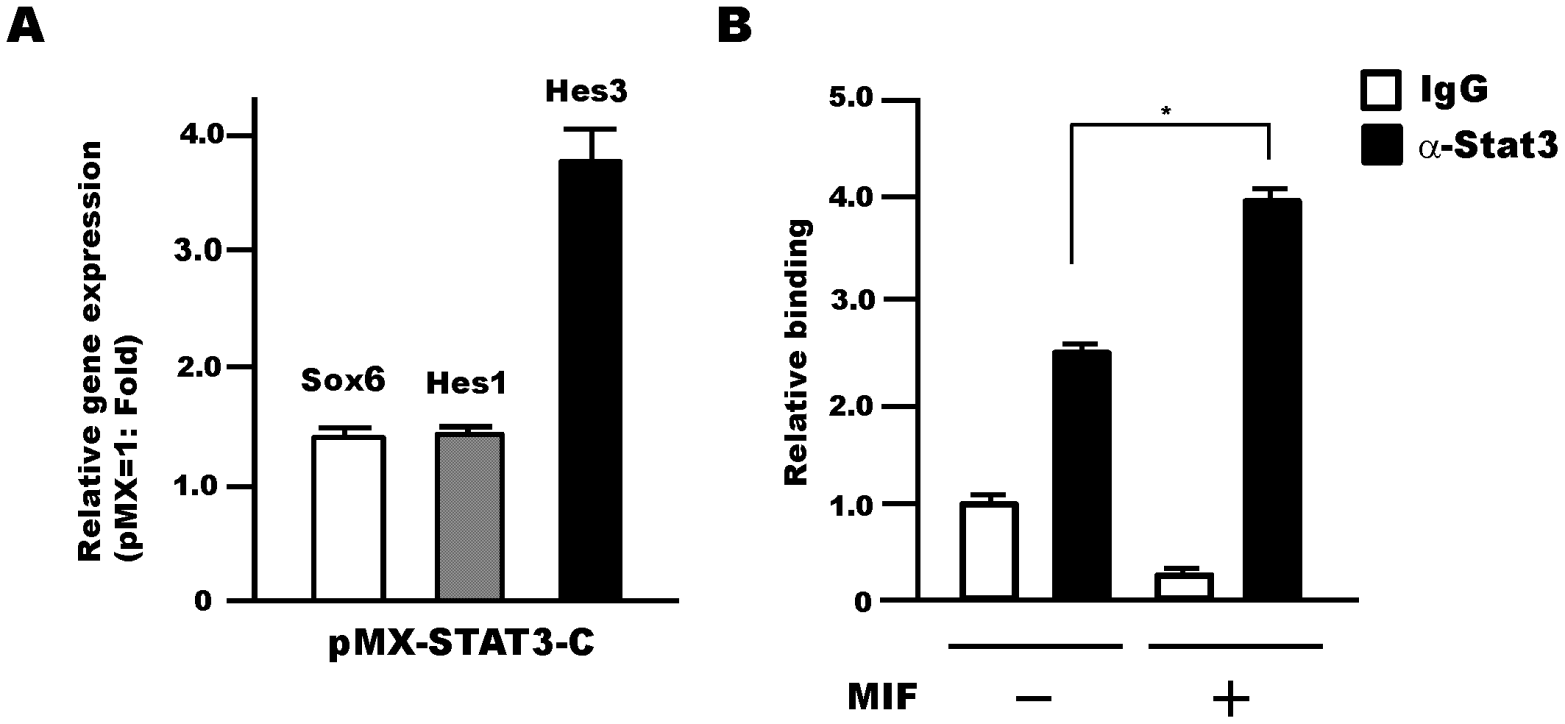
Stat3 is involved in the MIF-Sox6 signaling cascade in NSPCs. (A) Retrovirally-over expressed constitutively active Stat3 (pMX-Stat3-C) increased the gene expression levels of *Sox6, Hes1,* and *Hes3* in NSPCs compared to the expression levels in control retrovirus (pMX)-infected NSPCs 4 days after infection. Data are average of two independent experiments. (B) Changes in the binding ability of Stat3 to the *Sox6* promoter following MIF treatment (400 ng/ml, 5 days) in NSPCs were analyzed using the ChIP qPCR technique. Error bars indicate S.D. values; *P<0.05; Student’s *t*-test. Data are derived from three independent experiments.

We previously demonstrated that MIF treatment results in activation of the transcription factor Stat3 [16]. *Hes3* expression, which is activated by Stat3-pSer727 in NSPCs [25], was also shown to be upregulated by MIF [16]. Thus, we examined the changes in gene expression of *Sox6*, *Hes1*, and *Hes3* in NSPCs upon overexpression of constitutively active Stat3 in NSPCs, and found that the expression level of all genes was increased (Fig. 6A). Moreover, ChIP analysis showed an increase in Stat3 binding to the *Sox6* promoter following MIF treatment of NSPCs (Fig. 6B). These data thus suggest that Sox6 is upregulated by MIF signaling in NSPCs through binding of Stat3 to its promoter.

### MIF-mediated Up-regulation of Sox6 Supports the Self-renewal Ability of NSPCs

**Figure 7:**
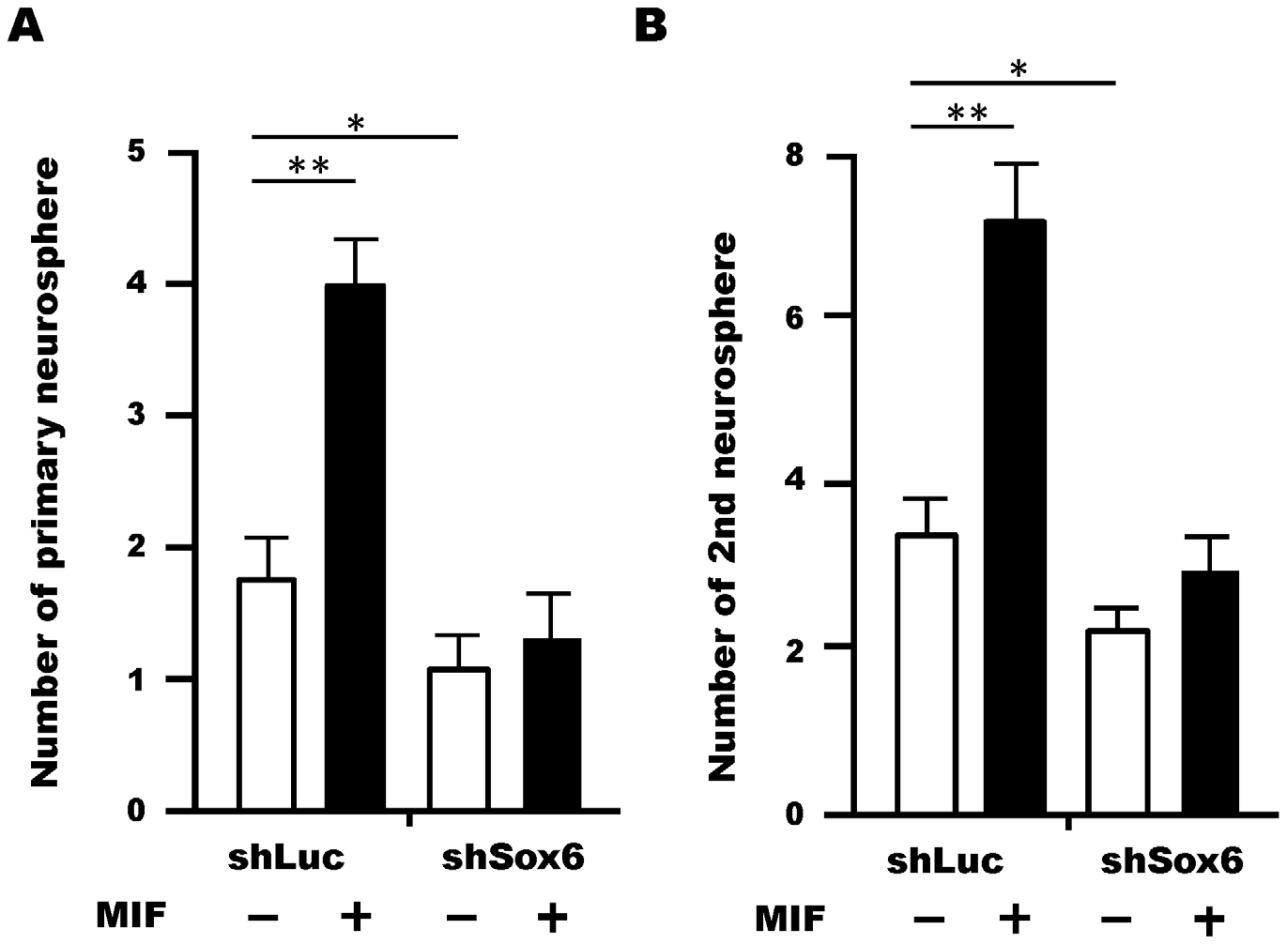
MIF regulated Sox6 can support cell survival and/or self-renewal ability in NSPCs. (A,B) Changes in the number of both primary (A) and secondary (B) neurospheres formed with or without MIF treatment were observed. The increase in neurosphere formation by MIF treatment (400 ng/ml) was disturbed by *Sox6* gene knockdown in both the primary and secondary neurosphere formation assays. All neurosphere formation assays were performed at a low cell density (1 cell/µl). Data are means S.D. values, average of three (A) or four (B) independent experiments; *P<0.05, **P<0.01 versus control; Student’s *t*-test.

To examine whether Sox6 plays a role in neurosphere formation in response to MIF, we performed neurosphere formation assays using cells treated with a retrovirus expressing Sox6-shRNA. While MIF treatment of control cells resulted in a larger number of primary (Fig. 7A) and secondary neurospheres (Fig. 7B), this effect was blunted by knockdown of Sox6, indicating that Sox6 is a downstream effector of MIF signaling in NSPCs. The same result was observed in a cell growth assay using lentivirally expressed *Sox6* shRNA in NSPCs (Fig. S7).

## Discussion

In the present study, we newly identified Sox6, which is expressed in the GE of the fetal mouse brain, as a maintenance factor for NSPCs. Moreover, we showed that *Sox6* gene expression is upregulated by Stat3 downstream of MIF, a signaling molecule that supports the proliferation and/or survival of murine NSPCs.

To determine whether MIF can regulate Sox genes to maintain NSPC stemness, we analyzed change in gene expression levels of Sox1, Sox2 and Sox6 in response to MIF treatment in NSPCs. Sox2 is known as a maintenance factor for NSPC stemness in fetal and adult mouse brains [3]. Interestingly, in this assay system, MIF treatment increased *Sox6*, but not *Sox1* or *Sox2* gene expression. Consistent with this finding, treatment of NSPCs with ISO-1, a MIF inhibitor, decreased *Sox6* gene expression, suggesting that Sox6 is a downstream molecule of MIF in NSPCs. However, it is possible that MIF may modulate *Sox1* or *Sox2* gene expression in other assay conditions and in NSPCs derived from different tissue types, including the cortex and spinal cord. Analysis by luciferase assay using a *Sox6* promoter also showed that *Sox6* gene transcription is upregulated by MIF in NSPCs, suggesting that *Sox6* is a direct target of MIF signaling in this system.

We demonstrated that Sox6 overexpression can increase the survival and self-renewal ability of NSPC, and that *Sox6* gene silencing in NSPCs has opposite effects. We observed an increase in *Bcl2* expression and Akt phosphorylation in response to MIF treatment, both of which play an important role in NSPC cell survival [16], further suggesting that Sox6 is a survival factor acting downstream of MIF in NSPCs. In contrast, a previous report showed that SOX6 overexpression decreased cell proliferation in INS-1E insulinoma cells and NIH3T3 cells [26], indicating the possibility that *Sox6* may regulate cell proliferation in a cell type-dependent manner. Furthermore, it is known that Sox proteins function in concert with partner proteins in a cell type-specific manners [27]. Thus, the identification of Sox6 partner proteins in NSPCs is a topic for future studies that would allow for an understanding of Sox6 protein mode of action.

We showed here that Sox6 is a maintenance factor for NSPCs in neurosphere-forming and differentiation assays *in vitro*, and observed up-regulation of *Hes1* gene expression upon Sox6 overexpression, as also seen upon MIF treatment. Hes1 is known as an important factor for NSPC maintenance [24]. Although Hes3 was also found to be up-regulated by MIF [16], *Hes1* has Sox6 binding elements in its promoter, while *Hes3* does not. Thus, Sox6 may possibly activate Hes1 with other transcription factors, as seen in chondrocytes [28], while Hes3 may be regulated directly by Stat3 without the intervention of Sox6. In previous reports, Sox6 was shown to support neurogenesis and gliogenesis and to inhibit terminal differentiation of oligodendrocytes [10], [11], [14]. Scheel et al have reported that Sox6 overexpression induces astrocytic differentiation from rat hippocampal NSPCs [14]. Thus, it may be important to examine Sox6 function in NSPCs derived from different species and tissue types (e.g. adult SVZ of forebrain, adult hippocampus, fetal cortex), especially if different NSPC culture methods are used (neurosphere floating culture VS adherent culture). Additionally, it would also be interesting to examine the regulation of Sox6 function by MIF in different types of tissue stem cells, including hair follicle stem cells [29], [30]. Moreover, Sox6 was reported to regulate the differentiation of neural progenitor cells into different neural cell types in the mouse embryonic brain *in vivo* [12], [13]. In those *in vivo* studies, the expression patterns of many transcription factors changed in the mouse embryonic GE in response to the loss of *Sox6*, showing that Sox6 may play a role in controlling the maintenance of stemness of NSPCs via or together with several transcription factors.

In our previous report [16] we found that although MIF induced the self-renewal ability of NSPCs, it did not change the cell fate of NSPCs *in vitro*, as there was no significant difference in the cell differentiation potential of NSPCs upon MIF treatment. However, in the present study, *Sox6* overexpression in NSPCs *in vitro* resulted in fewer differentiated cells belonging to three lineages. As *Sox6* is just one of multiple genes activated by MIF, changes in the cell fate of NSPCs by *Sox6* overexpression do not necessarily mirror changes induced by MIF treatment. Strong Sox6 activity may have been responsible for the decrease in differentiation markers in this system, which was not achieved in a previous study which examined the effects of MIF, a factor that lies upstream of Sox6.

The expression of SOX genes has been reported in many tumors [31]. We reported high expression of SOX genes in human gliomas in a previous report [15]. To date, high expression of *SOX6* in gliomas has been confirmed by *in silico* gene expression databases, including Oncomine (www.oncomine.org). We have observed higher levels of *SOX6* expression in glioma-initiating cells generated from glioma specimens compared to neural stem cells (Ohta et al., unpublished data). Thus, it may be important to analyze the detailed function of Sox6 in gliomas and glioma-initiating cells in the future.

In this study, we focused on Sox6 function in NSPCs derived from the GE of mouse embryonic brains *in* *vitro*, showing a new function of Sox6 as a maintenance factor of NSPCs stemness. Functional analyses of Sox6 in adult mouse NSPCs, and especially in human gliomas and glioma-initiating cells, will pave the way for evaluating Sox6 as a therapeutic target for many brain diseases.

## Supporting Information

Figure S1 **Self-renewal ability of Sox6 mutant NSPCs.** In the primary neurosphere formation assay, single dissociated cells from E14.5 GEs of mouse fetal brains taken from of Sox6 mutant and littermate controls were seeded onto a 96-well plate at a cell density of 10 cells/µl in the presence of EGF and FGF2. WT (n = 9), KO (n = 6). In the secondary neurosphere assay, primary neurospheres were dissociated into single cells and seeded onto a 96-well plate at a cell density of 10 cells/µl in the presence of EGF and FGF2. WT (n = 8), KO (n = 9). Error bars indicate S.D. values; **P<0.01 versus control; Student’s *t*-test.
(TIF)

Figure S2 **Expression of Sox6 in the gain and loss of function experiments in NSPCs.** (A) Western blot analysis shows Sox6 protein expression in NSPCs infected with retrovirus expressing GFP alone (Ctrl), or GFP and Sox6 (Sox6) 5 days after infection. (B) Retroviral Sox6-shRNA expression significantly reduced Sox6 protein expression in NSPCs 5 days after infection.
(TIF)

Figure S3 **Expression pattern analysis of SOX6 promoter-derived Venus expression cells in glioma cells and NSPCs.** (A) FACS analysis of Venus reporter expression under the control of the human *SOX6* promoter in human dermal cells (TIG118), human glioma cells (SF126, U87MG), and human NSPCs (NSP). (B) *SOX6* gene expression levels in TIG118, SF126, U87MG, and human NSPCs. (C) Immunostaining of Sox6 in mouse NSPCs infected with a lentivirus expressing the Venus reporter under the control of Sox6 showing co-localization of Sox6-positive cells and Venus- positive cells. Scale bar; 100 µm, 20 µm (enlarged image). Data are derived from three independent experiments.
(TIF)

Figure S4 **Identification of MIF-responsive elements in the SOX6 promoter.** (A) Schematic representation of A-box and B-box location in human *SOX6* gene promoter [21]. (B) Luciferase-reporter analysis of a region from the *SOX6* promoter (−517), and A-box and B-box tandem repeats in NSPCs, either with or without MIF treatment, 48 h after transfection. Relative luciferase activity was calculated by dividing the firefly luciferase activity of the constructs by the Renilla luciferase activity of the tyrosine kinase promoter, pRL-TK. Data show a representative data from three independent experiments. Error bars indicate S.D. values; *P<0.05, **P<0.01 versus control; Student’s *t*-test.
(TIF)

Figure S5 **Sox6 supports cell survival in NSPCs.** (A) Sox6 targeting using lentiviral shRNA significantly reduced NSPC growth compared to control shRNA, as assessed using a Cell Titer-Glo Assay Kit 4 days after infection. (B) Sox6 knockdown by lentvirally-expressed shRNA led to an increase in caspase 3/7 activity in NSPCs 4 days after infection. (C) *Sox6* gene expression in NSPCs infected with control lentivirus or lentivirus expressing Sox6-shRNA 4 days after infection. Data are derived from three independent experiments. Error bars indicate S.D. values; *P<0.05, **P<0.01 versus control; Student’s *t*-test.
(TIF)

Figure S6 **Differentiation potential of NSPCs derived from Sox6 knockout mice.** (A) Secondary neurospheres of Sox6 mutant and wild type were dissociated and cultured for 5DIV in the absence of growth factors. The differentiated cells were labeled with a neuronal marker (TuJ1), an astrocyte marker (GFAP), or an oligodendrocyte marker (CNPase) and counted. Data are averages of five independent experiments. Error bars indicate S.D. values; *P<0.05, **P<0.01 versus control; Student’s *t*-test. (B) Representative images of cells differentiated from Sox6 mutant and wild type neurospheres. Scale bar: 50 µm.
(TIF)

Figure S7 **MIF regulated Sox6 can support cell survival and/or proliferative ability in NSPCs.** Changes in cell number with or without MIF treatment (400 ng/ml) in NSPCs were observed using a CellTiter Glo Luminescent Cell kit. The increase in cell viability by MIF treatment was inhibited by lentiviral Sox6 gene knockdown 4 days after infection (n = 3). Error bars indicate S.D. values; *P<0.05, **P<0.01 versus control; Student’s *t*-test from three independent experiments.
(TIF)

Table S1 **Primer sequence.**
(DOC)
