# Supplementary figures and images for: Sox6 Up-Regulation by Macrophage Migration Inhibitory Factor Promotes Survival and Maintenance of Mouse Neural Stem/Progenitor Cells
Source: PLoS One. 2013 Sep 16;8(9):e74315. doi: 10.1371/journal.pone.0074315 (PMC3774630; doi:10.1371/journal.pone.0074315)

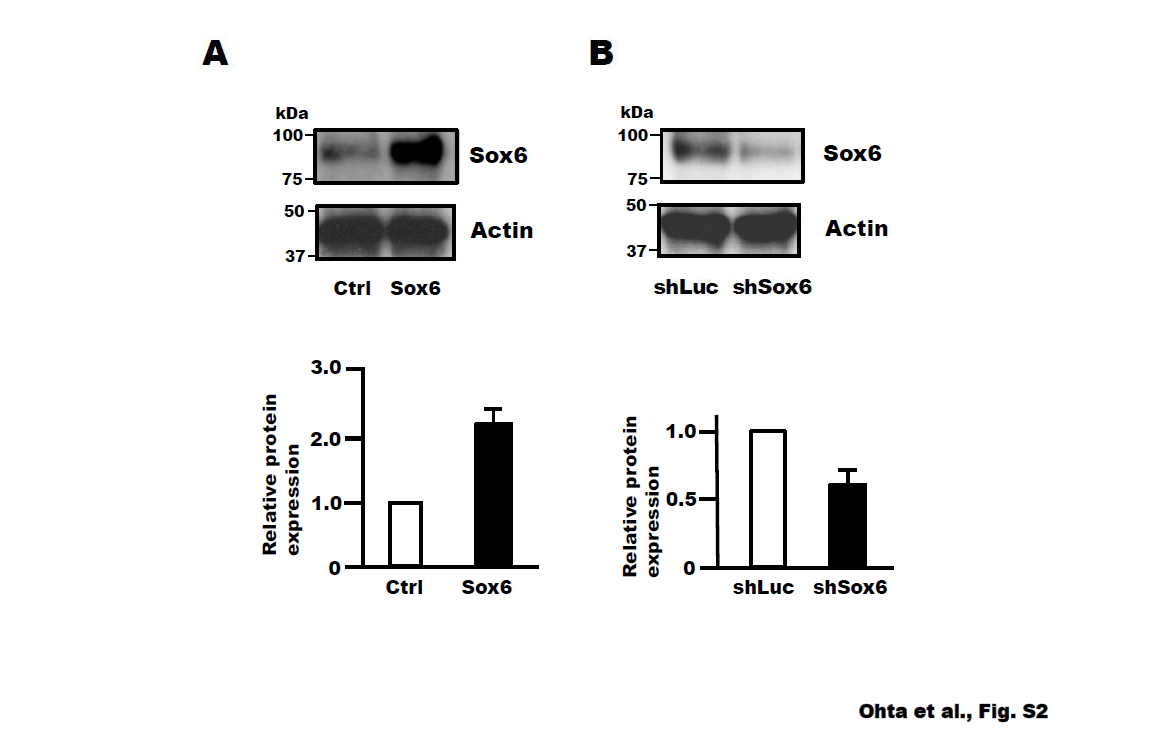

Supplement: Figure S2 — Expression of Sox6 in the gain and loss of function experiments in NSPCs. (A) Western blot analysis shows Sox6 protein expression in NSPCs infected with retrovirus expressing GFP alone (Ctrl), or GFP and Sox6 (Sox6) 5 days after infection. (B) Retroviral Sox6-shRNA expression significantly reduced Sox6 protein expression in NSPCs 5 days after infection. (TIF) [file pone.0074315.s002.tif]

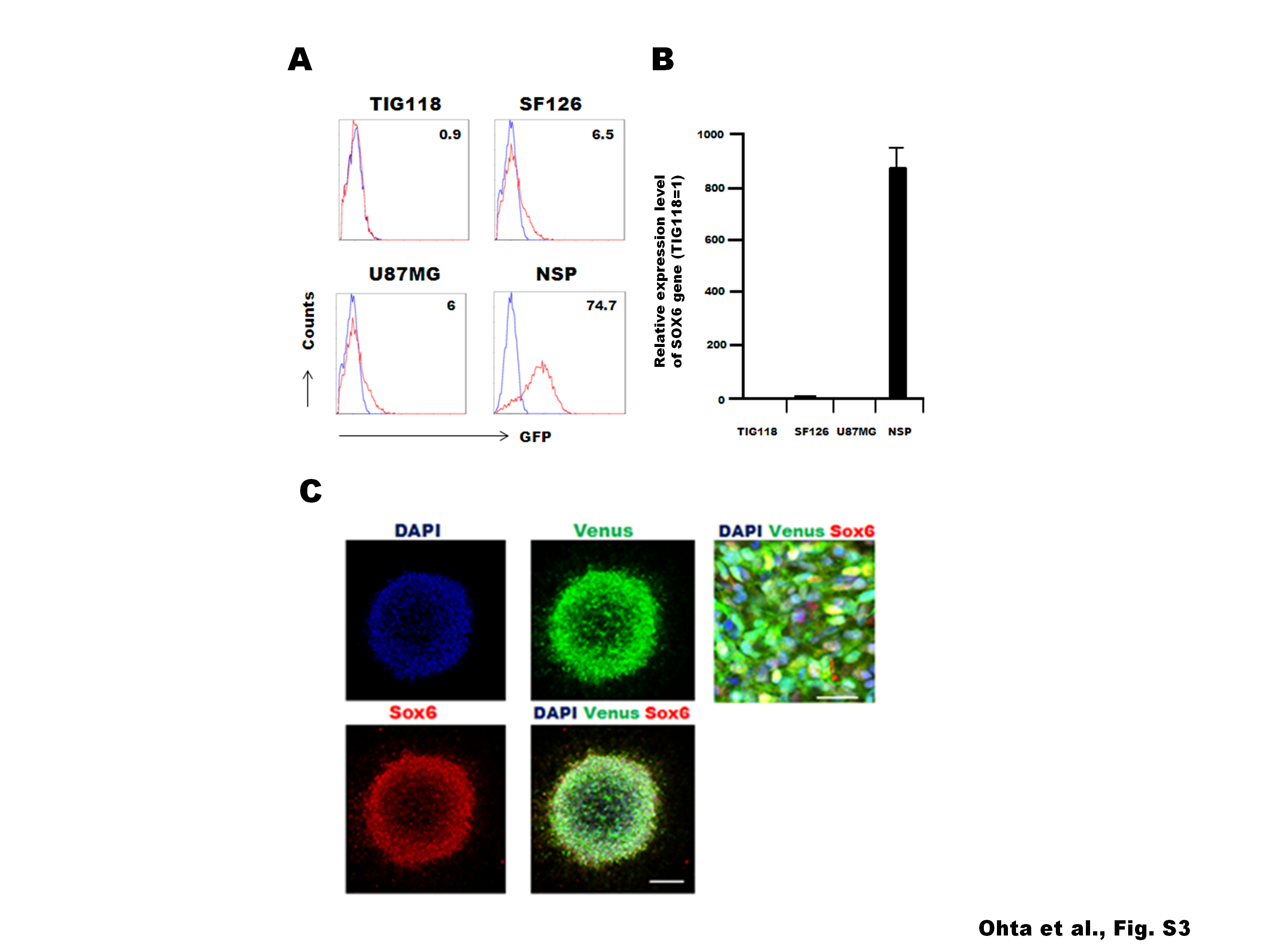

Supplement: Figure S3 — Expression pattern analysis of SOX6 promoter-derived Venus expression cells in glioma cells and NSPCs. (A) FACS analysis of Venus reporter expression under the control of the human SOX6 promoter in human dermal cells (TIG118), human glioma cells (SF126, U87MG), and human NSPCs (NSP). (B) SOX6 gene expression levels in TIG118, SF126, U87MG, and human NSPCs. (C) Immunostaining of Sox6 in mouse NSPCs infected with a lentivirus expressing the Venus reporter under the control of Sox6 showing co-localization of Sox6-positive cells and Venus- positive cells. Scale bar; 100 µm, 20 µm (enlarged image). Data are derived from three independent experiments. (TIF) [file pone.0074315.s003.tif]

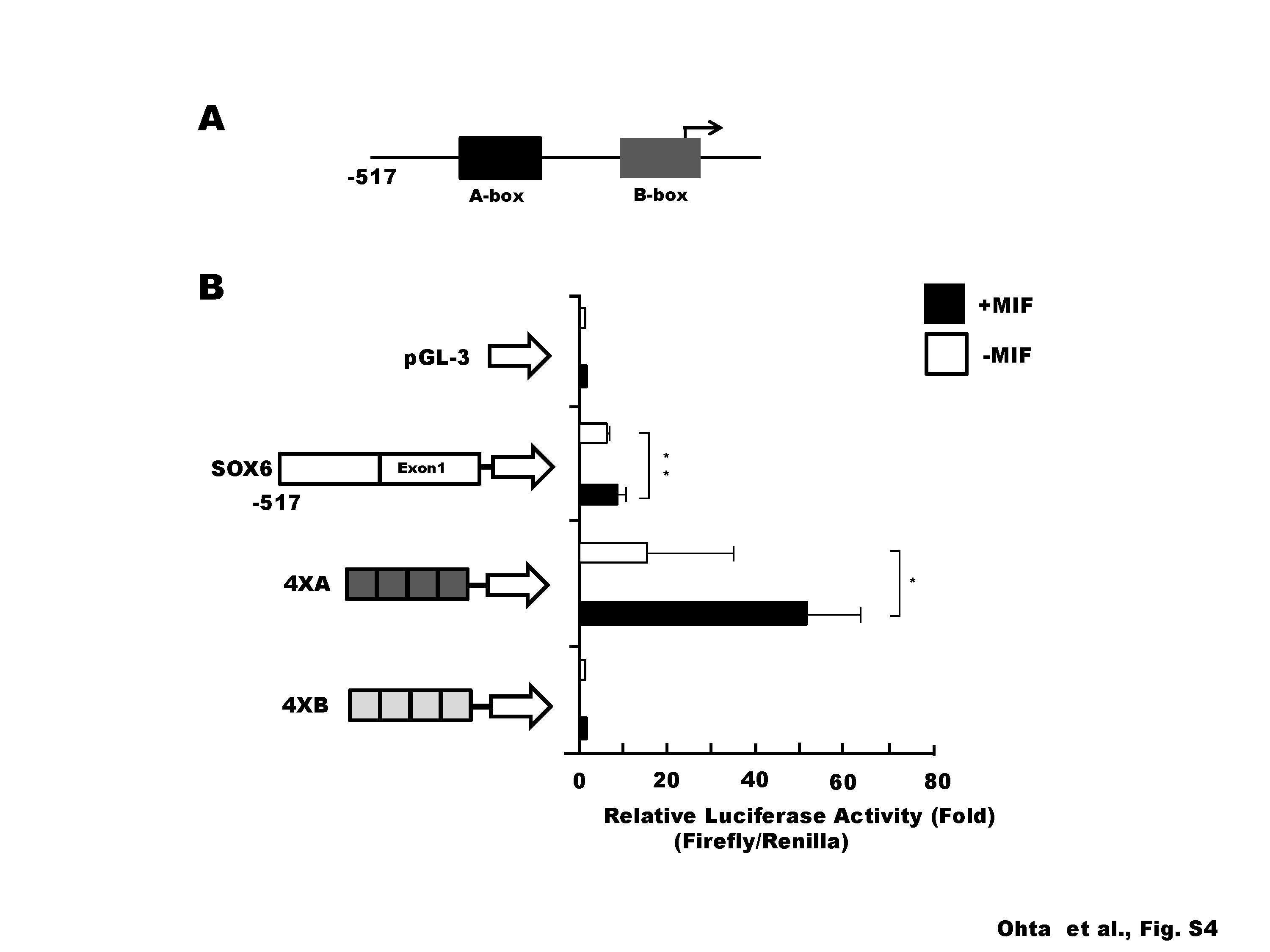

Supplement: Figure S4 — Identification of MIF-responsive elements in the SOX6 promoter. (A) Schematic representation of A-box and B-box location in human SOX6 gene promoter [21]. (B) Luciferase-reporter analysis of a region from the SOX6 promoter (−517), and A-box and B-box tandem repeats in NSPCs, either with or without MIF treatment, 48 h after transfection. Relative luciferase activity was calculated by dividing the firefly luciferase activity of the constructs by the Renilla luciferase activity of the tyrosine kinase promoter, pRL-TK. Data show a representative data from three independent experiments. Error bars indicate S.D. values; *P<0.05, **P<0.01 versus control; Student’s t-test. (TIF) [file pone.0074315.s004.tif]

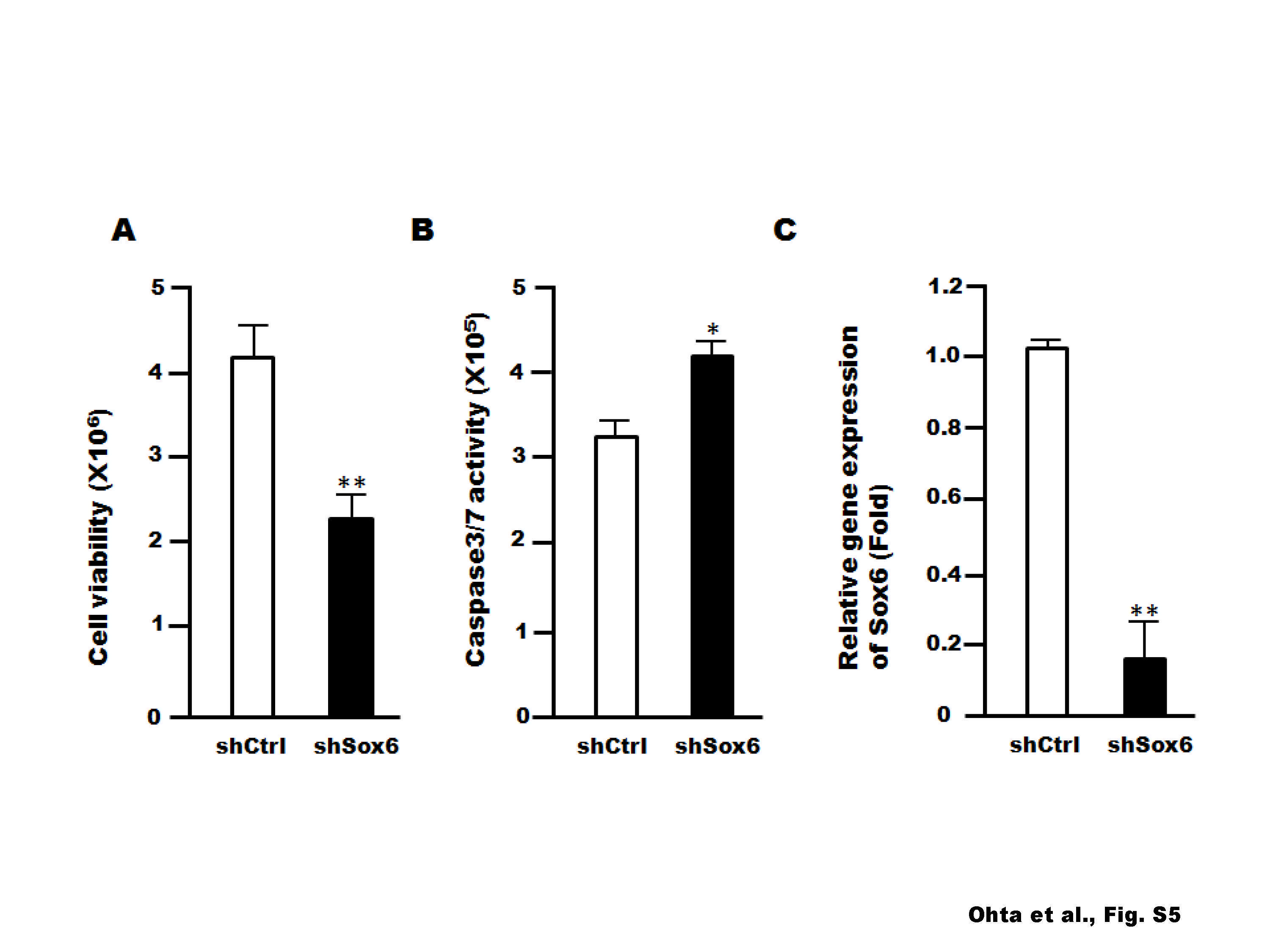

Supplement: Figure S5 — Sox6 supports cell survival in NSPCs. (A) Sox6 targeting using lentiviral shRNA significantly reduced NSPC growth compared to control shRNA, as assessed using a Cell Titer-Glo Assay Kit 4 days after infection. (B) Sox6 knockdown by lentvirally-expressed shRNA led to an increase in caspase 3/7 activity in NSPCs 4 days after infection. (C) Sox6 gene expression in NSPCs infected with control lentivirus or lentivirus expressing Sox6-shRNA 4 days after infection. Data are derived from three independent experiments. Error bars indicate S.D. values; *P<0.05, **P<0.01 versus control; Student’s t-test. (TIF) [file pone.0074315.s005.tif]

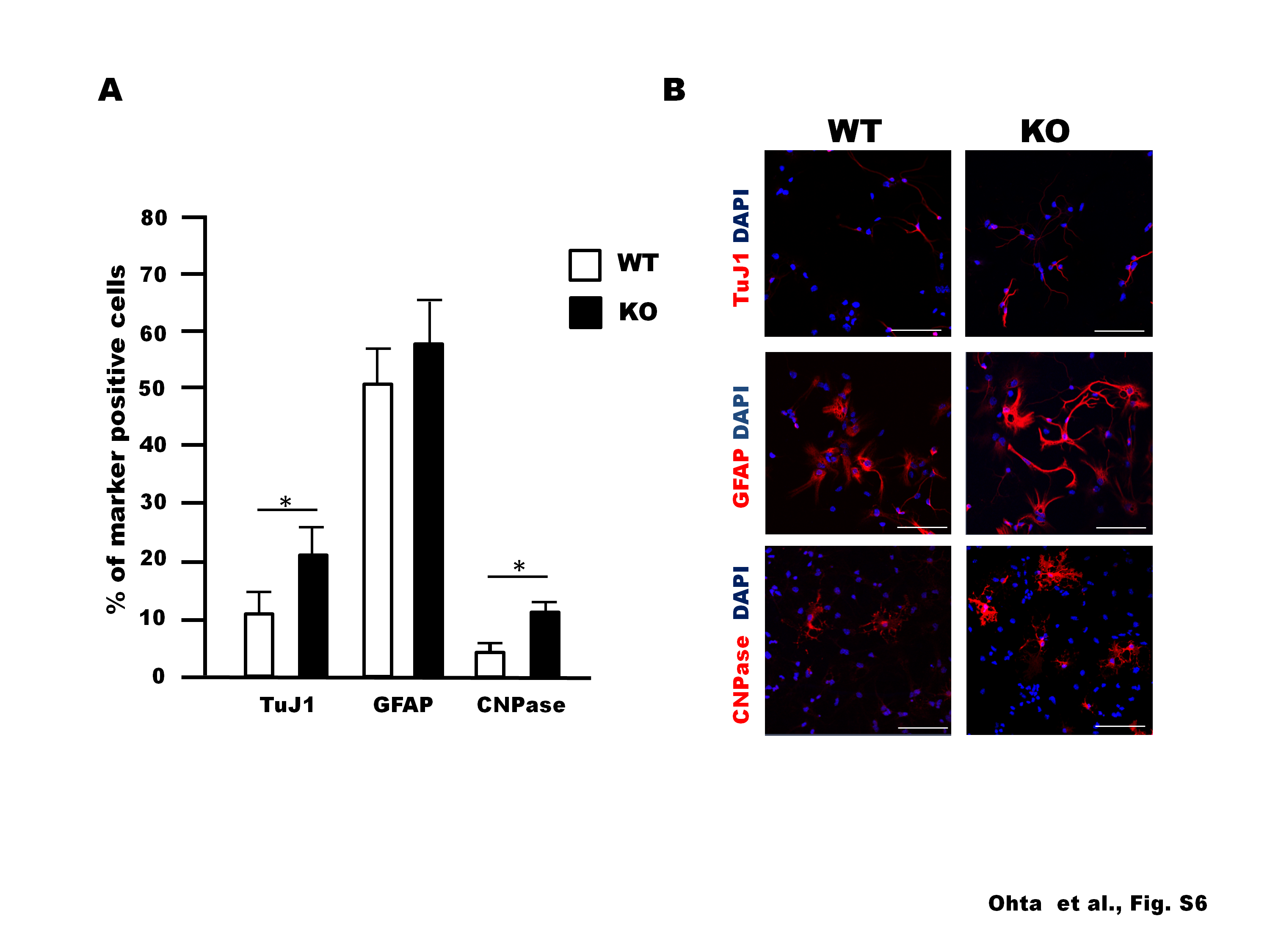

Supplement: Figure S6 — Differentiation potential of NSPCs derived from Sox6 knockout mice. (A) Secondary neurospheres of Sox6 mutant and wild type were dissociated and cultured for 5DIV in the absence of growth factors. The differentiated cells were labeled with a neuronal marker (TuJ1), an astrocyte marker (GFAP), or an oligodendrocyte marker (CNPase) and counted. Data are averages of five independent experiments. Error bars indicate S.D. values; *P<0.05, **P<0.01 versus control; Student’s t-test. (B) Representative images of cells differentiated from Sox6 mutant and wild type neurospheres. Scale bar: 50 µm. (TIF) [file pone.0074315.s006.tif]
