# Supplementary material for: Sox6 Up-Regulation by Macrophage Migration Inhibitory Factor Promotes Survival and Maintenance of Mouse Neural Stem/Progenitor Cells
Source: PLoS One. 2013 Sep 16;8(9):e74315. doi: 10.1371/journal.pone.0074315 (PMC3774630; doi:10.1371/journal.pone.0074315)
Supplement: Table S1 — Primer sequence. (DOC) [file pone.0074315.s008.doc]

**Table S**1 Primer sequence

| **Target** | **Primer name** | **Sequence** |
| --- | --- | --- |
| Sox1 | Sox1 Fwd  Sox1 Rev | AGATGCACAACTCGGAGATCAG  GAGTACTTGTCCTTCTTGAGCAGC |
| Sox2 | Sox2 Fwd  Sox2 Rev | TTAACGCAAAAACCGTGATG  GAAGCGCCTAACGTACCACT |
| Sox5 | Sox5 Fwd  Sox5 Rev | TGGAGATTCTGACGGAAGCG  CTTGTCCCGCAATGTGGTT |
| Sox6  (Mouse) | mSox6 Fwd  mSox6 Rev | TTGGGGAGTACAAGCAACTGATGC ATCTGAGGTGATGGTGTGGTCGTT |
| SOX6 (Human) | hSOX6 Fwd  hSOX6 Rev | TAAGCAACTGATGAGGTCTC  AGGCGATGGTGTGGTAGTT |
| Nestin | Nestin Fwd  Nestin Rev | TGAAGCACTGGGAAGAGTAG  TAACTCATCTGCCTCACTGTC |
| Musashi-1 | Msi-1Fwd  Msi-1 Rev | GAGACTGACGCGCCCCAGCC  CGCCTGGTCCATGAAAGTGACG |
| Bcl-2 | Bcl-2 Fwd  Bcl-2Rev | ATCTTCTCCTTCCAGCCTGA TCAGTCATCCACAGGGCGAT |
